# Oral contraceptive use increases bone density and reduces the risk of osteoporosis

**DOI:** 10.1007/s10654-025-01273-2

**Published:** 2025-07-09

**Authors:** Emma L. Ivansson, Therese Johansson, Torgny Karlsson, Åsa Johansson

**Affiliations:** 1 Department of Immunology, Genetics and Pathology, Science for Life Laboratory, Uppsala University, Uppsala, Sweden; 2 KTH, Science for Life Laboratory, Stockholm, Sweden; 3 Garvan Institute of Medical Research, Sidney, Australia

**Keywords:** Oral contraceptives, Bone mineral density, Osteoporosis, Postmenopausal, Estrogen

## Abstract

Osteoporotic fractures, largely resulting from reduced estrogen levels after menopause and subsequent bone loss, are a leading cause of disability among older women. Although oral contraceptive pills (OCPs) contain estrogen, their long-term impact on bone health and osteoporosis risk remain uncertain. Here, we assessed the effect of OCP use on bone mineral density (BMD) and osteoporosis using data from 257,185 women from the UK Biobank, born 1936–1970. Time-dependent Cox regression was used to estimate adjusted hazard ratios (HRs) and 95% confidence intervals (CI) for osteoporosis, while multivariable linear regression was used to assess the effect of OCP use on BMD, measured as T-scores in standard deviation units based on quantitative ultrasound of the calcaneus. By the end of follow-up in 2020, 7.6% of the participants had received an osteoporosis diagnosis. The rate of osteoporosis was lower among ever OCP users (HR = 0.86; 95% CI 0.83–0.89; *P* = 2.8 × 10^−17^). OCP use was also associated with a higher BMD T-score (0.052; 0.038–0.067; *P* = 2.1 × 10^−12^) with an increasing effect with longer use. Use of OCPs for 0–1 years had no significant effect on BMD (*P* = 0.081). However, longer durations were associated with increased BMD T-scores compared to never users: 2–5 years (0.046; 0.027–0.065, *P* = 2.2 × 10^−6^), 6–10 years (0.062; 0.043–0.080; *P* = 3.5 × 10^−12^), 11–15 years (0.062; 0.042–0.081; *P* = 3.2 × 10^−12^) and 16 + years (0.064; 0.044–0.083; *P* = 1.2 × 10^−10^). We found prior OCP use to be associated with higher BMD and a reduced risk of osteoporosis, potentially offering long-term benefits and suggesting that OCP use could reduce osteoporotic complications in older women.

**Supplementary Information:**

The online version contains supplementary material available at 10.1007/s10654-025-01273-2.

## Introduction

Osteoporosis is a common skeletal disorder characterized by compromised bone strength, predisposing a person to an increased risk of fracture. Bone mineral density (BMD) accounts for about 70% of bone strength and is often used as a proxy for bone strength [1]. The World Health Organization defines osteoporosis as bone density 2.5 standard deviations below the mean for young white adult women [2]. In clinical practice, however, postmenopausal osteoporosis may be treated based on a history of fragility fractures without assessing BMD [3, 4]. Osteoporotic fragility fractures cause a greater hospitalization burden and hospital costs than myocardial infarction, stroke or breast cancer in US women 55 years and older [5], and lead to a significant rise in mortality [6, 7]. Well-established risk factors for decreased BMD and increased risk of osteoporosis include low body mass index (BMI) [8] and smoking [9]. During menopause, loss in bone mass [10] and strength [11] accelerates, presumably due to a reduction in levels of estradiol [12], and increasing age is the therefore the major risk factor.

Several factors have been suggested to promote bone health and reduce the risk of osteoporosis. These include a diet rich in calcium, and vitamin D, regular physical activity, and the use of hormone replacement therapy (HRT) with estrogen for postmenopausal women [13, 14]. In particular, the use of HRT, has been found to increase BMD and reduce fracture risk [15]. The link between estrogen deficiency and osteoporosis was pointed out already in 1941 [16] and estrogen is considered a key regulator of bone metabolism [17, 18]. Worldwide, around 16% of women age 15–49 years (151 million) were using oral contraceptive pills (OCP) containing exogenous hormones in 2019 [13] and 80% of women in reproductive age in Europe and North America will at some point use OCP [14]. However, the effect on bone health is not well characterized. Several smaller studies aimed to estimate the effect of OCP on bone health suggest a potential positive effect of OCP on BMD in perimenopausal women, while findings in premenopausal women are contradictory, as reviewed in [19–21]. Most previous studies evaluating the risk of OCP use on BMD, osteoporosis, or fractures have been limited by the long follow-up time required for women to develop osteoporosis and/or the lack of BMD measurements in population based studies. However, previous studies have suggested, for example, a 25% reduced risk of hip fractures, in previous users of OCP [22].

Given the well-established link between estrogen and bone health, along with findings from several small and underpowered studies, we hypothesize that OCP use is associated with higher BMD and a lower risk of osteoporosis in later life. To test this hypothesis and to to expand the current knowledge on the effects of OCP use on later-life bone health, we here examine two skeletal health outcomes: quantitative BMD measurements and osteoporosis diagnoses, in 257 185 white female participants from the UK Biobank (UKB) cohort.

## Methods

### Study population

UKB is a population-based cohort including more than 500 000 United Kingdom (UK) residents [23], aged 37–72 years at the recruitment in 2006–2010. Baseline assessments occurred at enrollment across 22 UK centers and included questionnaires and interviews on medical history, lifestyle and previous exposures as well as physical health measures. Diagnoses were self-reported during initial assessment and collected (retrospectively and prospectively) from hospital records, cause of death registers and primary care data [24]. The UKB study has received general ethical approval from the National Research Ethics Committee (REC reference 11/NW/0382). The present use of UKB data was specifically approved under application number 41,143, with data released on February 4, 2021. In addition, the analysis conducted in the current investigation was approved by the Swedish Ethical Review Authority (dnr: 2020–04415).

### Study outcomes

Osteoporosis diagnoses were retrieved from a curated data set (UKB field id: 131962 and 131965) comprising the date of the first osteoporosis diagnosis for each participant. These diagnoses were based on self-reported medical history (collected through a verbal-interview with a trained nurse at the visit to the UKB assessment center) in combination with data from national health registries, including primary care, hospital admissions and cause-of death records until 2020, mapped to the ICD-10 codes: M80 (osteoporosis with pathological fracture) and M81 (osteoporosis without pathological fracture). The BMD measures were not used to define osteoporosis diagnoses in the cohort. BMD, based on quantitative ultrasound (QUS) measurement of the calcaneus (heel bone), was determined at recruitment for the majority of the UKB participants (57%). BMD is reported as a T-score, which compares the participant’s bone density with that normally expected in someone of the same sex. BMD is therefore a continuous trait, expressed in standard deviations (SD).

### Exposure to OCP and covariates

Information on OCP use, including age at initiating and discontinuing exposure, was obtained from touchscreen questionnaires during the initial assessment. The relevant questions include: “Have you ever taken the contraceptive pill?“, “About how old were you when you first went on the contraceptive pill?”, and “How old were you when you last used the contraceptive pill?”. For participants who reported that they were still using OCP at the recruitment, the age of last use was set to the age at the recruitment. The difference between the age of last use and the age of first use was used as a proxy for *duration* of OCP use. For women who were current users at the time of assessment (when BMD measures were taken), the difference between the age at assessment and the age of first use was used as a proxy for the duration of OCP use. Covariates for the analysis include age, year of birth, menopausal status (premenopausal, postmenopausal, uncertain), reproductive surgeries (hysterectomy yes/no, bilateral oophorectomy yes/no), number of life births, BMI (as a continuous variable) and current smoking (yes/no). Covariate information used in the present analysis was obtained at the time of recruitment to the UKB, which is also when BMD measurements were conducted.

### Inclusion and exclusion criteria

The UKB includes data from 273,375 women, and in this study, we included 257,185 women who self-identified as white Irish, white British, or other white backgrounds. Women with missing information on any of the covariates were excluded from all analyses. For osteoporosis, we used time-dependent Cox regression analyses, which require information on the age at initiation and discontinuation of OCP. In these analyses, participants were excluded if they lacked information on the age when they started using OCPs and/or the age of last use, resulting in 184,291 participants in the adjusted time-dependent Cox regression analyses.

For the BMD analyses, the cohort was limited to women who had undergone quantitative ultrasound (QUS) of the calcaneus (heel bone), resulting in 146,012 women (Supplementary Table S1). Women were first classified as either never users or ever users. Women who did not provide information on OCP use were excluded, resulting in 143 283 participants in the multivariate linear regression analyses. For the duration of use analyses, women who lacked information to estimate the duration of use were also excluded, resulting in 130 301 in the duration-stratified multivariate linear regression analyses.

### Statistical analysis

All statistical analyses was carried out in R version 4.1.1. Chi-square tests (frequency) and t-tests (mean) were used to evaluate overall differences between subgroups. Participants lacking complete information on outcome, exposure and covariates were excluded from the analysis.

When analysing osteoporosis, a prospective study design was employed, and data were analysed using time-dependent Cox regression. This approach was used regardless of whether the outcome data were collected retrospectively (e.g., self-reported at the time of recruitment to the UKB) or from register-based diagnoses including events after the recruitment. We compiled a data frame that included the age at which changes in exposures occurred (i.e., when women started and stopped using OCPs) and age at which changes time-varying covariates occurred. Additionally, the data frame included whether participants had received an osteoporosis diagnosis by the end of the follow-up, and if so, the age at which they were first diagnosed with osteoporosis. To assess the effect of OCP use on osteoporosis diagnosis, adjusted hazard ratios (HRs) were estimated using time-dependent Cox proportional hazards models. Age was used as the primary time scale [25], as this has been suggested to be the optimal way to account for age-related confounding, especially for outcomes such as osteoporosis, which increase sharply with age.

Since OCP use varies over time, it was modelled as a time-varying exposure using Cox models for counting processes [26]. In this framework, women were classified as non-users before initiating OCP use, current users during active use, and previous users after discontinuation. Non-users served as the reference group in the analyses. A time-varying representation of OCP use avoids misclassification of users’ survival time before OCP initiation and defines a natural start of follow-up for never users [26, 27]. This approach accurately represents the OCP status and classifies the “event-free” person-time of OCP users before their age at initiation as the unexposed follow-up time [28]. Note that age is naturally adjusted for non-parametrically, as age is used as the primary timescale in the Cox regression modelling. Results are presented as relative change in hazard rate ratios between current OCP users and never users, or previous users and never users. In the Cox regression, smoking and menopause were also modelled as time-varying covariates, while other covariates were treated as time-fixed.

We used the coxph () function from the R *survival* package, applying a counting process approach to appropriately handle time-varying exposures and covariates. This method allows individuals to enter follow-up at any time point, with exposure status updated accordingly. Women were followed until the first diagnosis of osteoporosis or the end of study follow-up in 2020, whichever occurred first.

The analyses of BMD were performed using a retrospective study design and multiple linear regression with OCP first analyzed as a binary exposure (ever use vs. never use). Further analysis stratified the duration of OCP use into different duration intervals: never, $\:\le\:$1 year, 2–5 years, 6–10 years, 11–15 years and > 15 years. Duration groups were compared with never users as the reference using linear regression. Sensitivity analyses were performed in premenopausal, postmenopausal and women of uncertain menopausal status separately. To evaluate the effect of initiating OCP use in adolescence, we stratified women based on age at OCP initiation, those who started before age 18 and those who started at age 18 or older and compared both groups to never-users. Results were presented as differences in BMD in units of T-score in standard deviation units.

## Results

### Baseline characteristics and OCP use in the UKB women included in the study

**Fig. 1:**
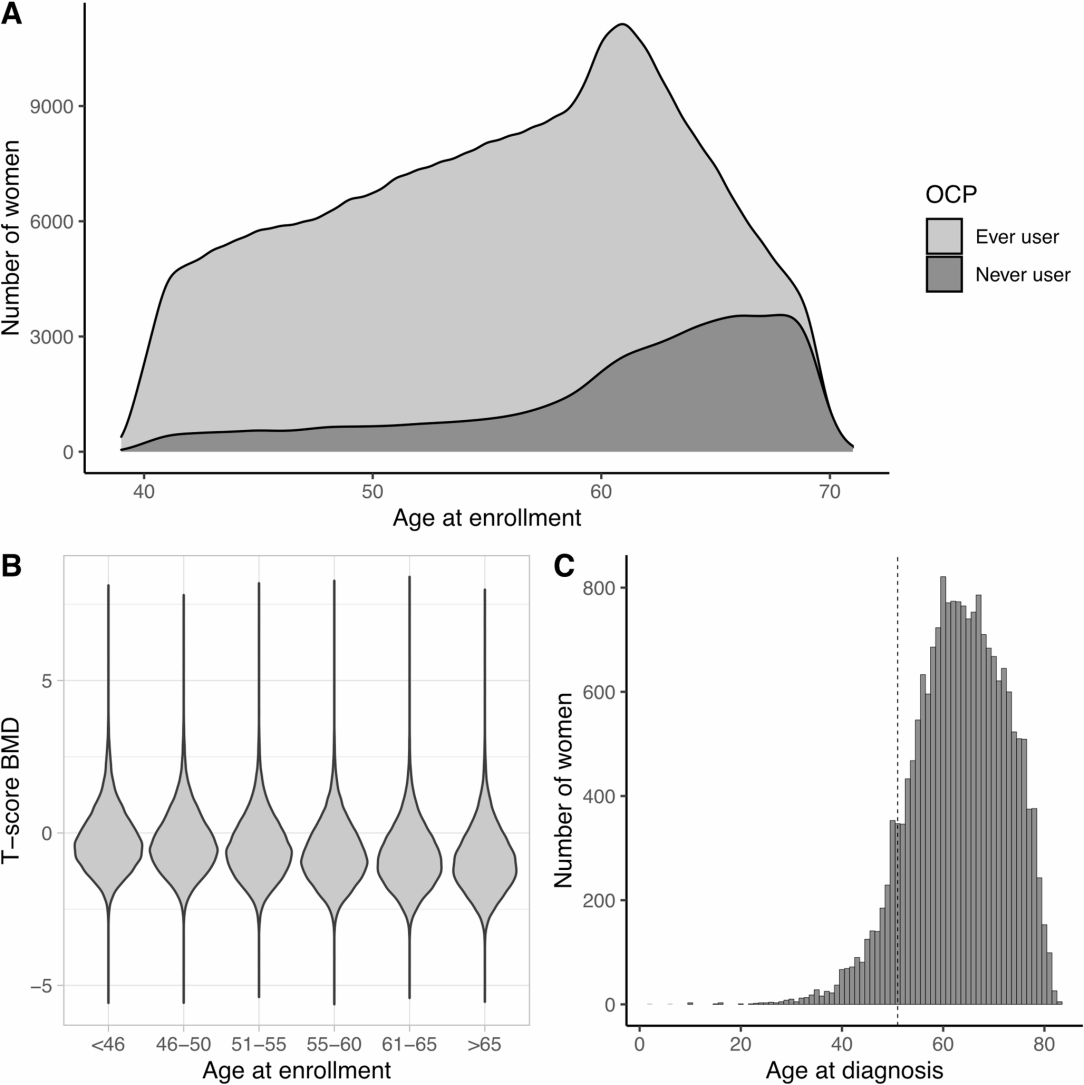
Age, BMD T-scores and osteoporosis diagnoses in UKB cohort. **A** Age at recruitment (initial assessment when BMD was measured) in UKB for women reporting that they had ever used or had never used OCP. **B** BMD T-scores from ultrasound measurement of the heel by age groups, with measurements taken at enrollment. **C** Number of osteoporosis cases by age at diagnosis. The dashed line represents the average age at menopause in UK women

A total of 257 185 women fulfilled the inclusion criteria (Table 1). The majority (61.3%) were postmenopausal, 23.6% were premenopausal and 15.8% were uncertain of their menopausal status when enrolled in the UKB study. Most women, (82%) reported that they had ever used OCP. Among these, 73% provided information on the age they initiated use and the age they last used OCP and the median age for first use was 21 years. A total of 20,130 women used OCPs for less than one year, 38,159 for between two and five years, 47,287 for six to ten years, 35,068 for eleven to fifteen years, and 46,401 used OCPs for more than fifteen years. On average, ever users were younger compared to never users (Table 1) and most women, regardless of age at recruitment, had at some point used OCP (Fig. 1A).

**Table 1:** Baseline characteristics of study population collected at the initial assessment (2006–2010), and osteoporosis diagnoses recorded until the end of 2020

	Full dataset	Stratified by OCP user status	
		*Ever users*	*Never users*
Total n (%)	257 185 (100%)	210 437 (82.0%)	46 215 (18.0%)
Age at recruitment range (mean)	39–71 (56.6)	40–70 (55.6)	39–71 (60.8)
Year of birth range (mean)	1936–1970 (1951)	1936–1970 (1952)	1936–1970 (1947)
Menopausal status n (%)			
Pre menopause	58 173 (23.6%)	52 620 (26.2%)	5 460 (12.1%)
Post menopause	157 722 (61.3%)	123 621 (58.7%)	33 837 (73.2%)
Uncertain menopause	40 708 (15.8%)	33 864 (16.1%)	6 742 (14.6%)
Missing data	300 (0.12%)	118 (0.06%)	111 (0.2%)
Hysterectomy	48 536 (18.9%)	37 785 (18.0%)	10 641 (23.0%)
Bilateral oophorectomy	20 752 (8.1%)	16 054 (7.6%)	4 653 (10.1%)
Number of life births range (mean)	0–22 (1.8)	0–22 (1.8)	0–22 (1.8)
BMI			
Range (mean)	12.1–74.7 (27.0)	12.1–74.7 (27.0)	12.7–67.2 (27.4)
Underweight (BMI < 18.5) n (%)	1 938 (0.8%)	1 491 (0.7%)	443 (1.0%)
Normal weight (18.5 < BMI < 25) n (%)	100 653 (39.3%)	84 062 (40.1%)	16 403 (35.5%)
Overweight (25 < BMI < 30) n (%)	94 184 (36.8%)	76 673 (36.6%)	17 323 (37.5%)
Obese (BMI > 30) n (%)	59 369 (23.2%)	47 421 (22.6%)	11 813 (25.6%)
Missing data n (%)	1 041	790	233
Current smoking n (%)	23 086 (9.0%)	19 756 (9.4%)	3 265 (7.1%)
Missing data	143	109	32
Calcaneal BMD n (%) ^a^	146 012 (56.8%)	119 464 (56.8%)	26 263 (56.8%)
T-score mean	−0.58	−0.55	−0.73
Osteoporosis diagnosis ^b^	19 485 (7.6%)	14 250 (6.8%)	5 176 (11.2%)

^a^Assessed by QUS at the time of enrollment in UKB

^b^Compiled from self-reported data and registry data, not based on the BMD recorded by UKB. Encompassing osteoporotic diagnoses with and without concurrent fragility fracture

### BMD T-scores among study participants

BMD was measured at enrollment to UKB for 146 012 (56.8%) of the participants, with similar proportions among ever and never users of OCP (Table 1). There was a clear trend for decreasing BMD with increasing age (Fig. 1B), *p* < 0.001. The baseline characteristics including the frequency of participants being diagnosed with osteoporosis were very similar between the full data set and the subset with available BMD data (Table S1). A total of 19 485 (7.6%) participants were diagnosed with osteoporosis either before or after enrollment (Table 1). Most osteoporosis diagnoses occurred after age 51 (Fig. 1C), which is the average age at menopause in the UK according to the NHS [29]. In line with never users being on average older, osteoporosis was more common in the group who had never used OCP (11.2% v 6.8%, *p* < 0.001).

### BMD T-scores are higher with previous OCP use

Ever using OCP was associated with a 0.052 (95% CI: 0.038–0.067) higher BMD T-score at enrolment (Table 2). The corresponding estimate from the sensitivity analysis was similar in the postmenopausal subset, 0.054 (0.036–0.071) and in the subset with uncertain menopausal status an even stronger effect was noted, 0.096 (0.057–0.14), but no significant effect was seen in the women who were still premenopausal at enrollment. Stratification of users based on age of initiation with the first quartile as cut-off yielded 59 389 women who started using OCP at age 18 or younger and 143 569 women who started using OCP when most bone mass has been gained, at age 19 or later. The estimates for these strata were 0.047 (0.028–0.067) and 0.055 (0.040–0.070).

**Table 2:** Effect of OCP ever use on BMD (T-score units). Regression coefficients (estimates) and p-values from multiple linear regression analysis are shown. In all analyses, ever users are compared to never users and the outcome is the measured BMD at baseline (Table 1)

	Estimate (95% CI)	*N*	*p*-value
Full dataset^a^	0.052 (0.038–0.067)	143 283	2.1e-12
Premenopausal^b^	−0.017 (−0.056–0.021)	33 481	0.38
Postmenopausal^b^	0.054 (0.036–0.071)	87 576	1.2e-09
Uncertain menopausal status^b^	0.096 (0.057–0.14)	22 226	1.7e-06

*N* number of participants with complete covariate information and thereby included in each analysis

^a^ Model adjusted for age, year of birth, BMI, smoking status, menopausal status, hysterectomy, bilateral oophorectomy and parity

^b^ Model adjusted for age, year of birth, BMI, smoking status, hysterectomy, bilateral oophorectomy and parity

#### Long-term OCP use is more strongly associated with increased BMD T-scores

**Fig. 2:**
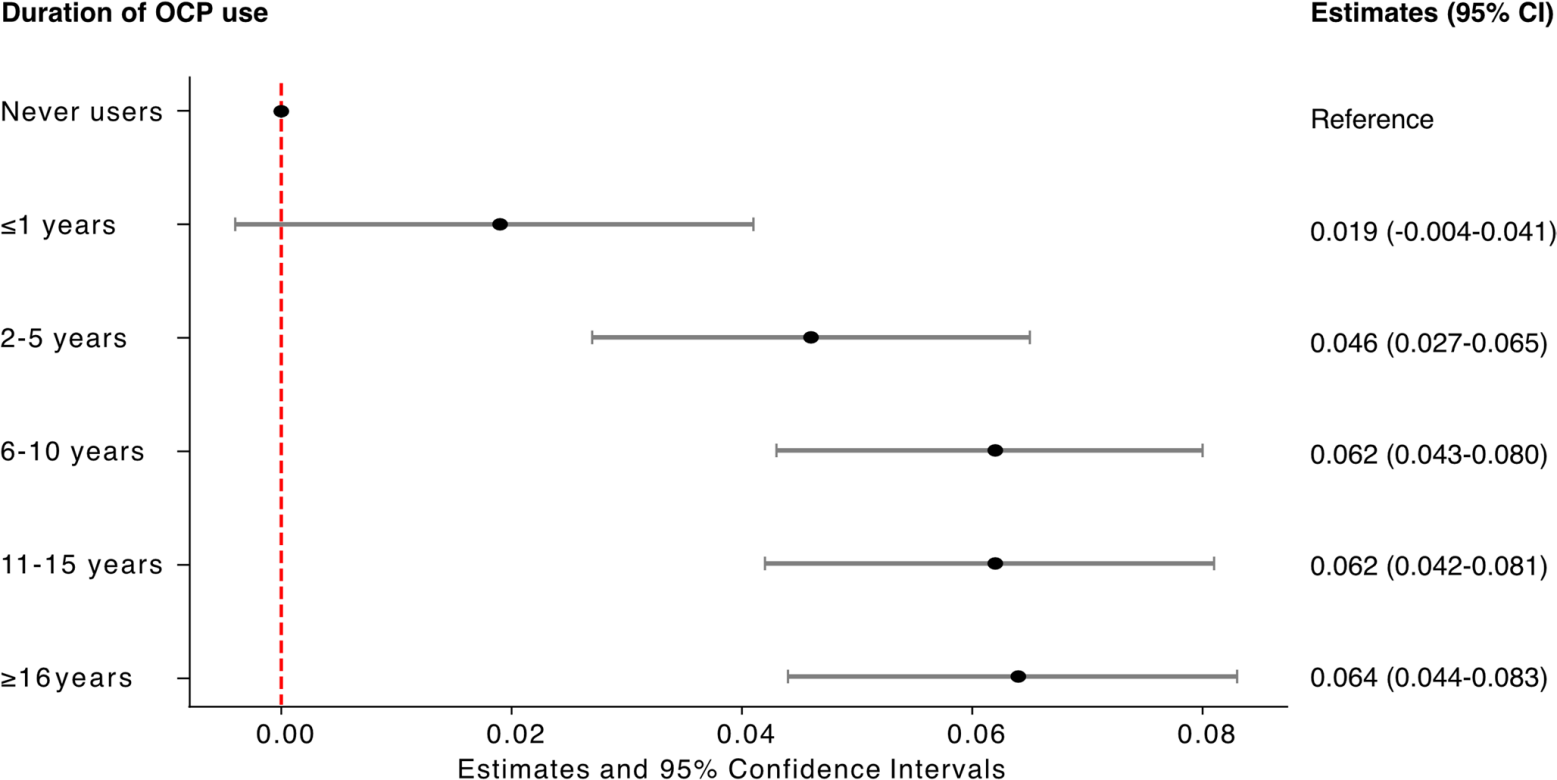
Estimates and 95% confidence intervals (CI) for duration of OCP use on average BMD T-scores. Users were stratified based on the duration of use: 1 year or less, 2–5 years, 6–10 years, 11–15, 16 years or more, and compared to never users as reference

There was no significant effect of short-term use (0–1 years) on BMD (Fig. 2). However, a longer duration was associated with increased BMD T-score compared to never users. A duration of 2–5 years yielded an estimate of 0.046 (0.027–0.065) and 6–10 years a higher estimate of 0.062 (0.043–0.080). The most extended duration, 16 years or more, provided the largest estimate 0.064 (0.044–0.083), but most of the increase was evident after 6–10 years of use and the extra effect associated with prolonged use after 10 years was negligible (Fig. 2).

### Risk of osteoporosis later in life is lower in OCP users

For osteoporosis, all diagnoses up until 2020 were included in the analyses, resulting in 19 485 women with an osteoporosis diagnosis (Table 3). After excluding participants with lack of full information on all covariates and exposure, 17,773 women with a diagnosis remained for the adjusted analyses. We modelled OCP use as a time-varying exposure with three levels: never user (including the period before initiating OCP use in ever users), during use, and previous use (after discontinuation). We observed no significant difference, HR = 1.00 (0.91–1.08) in the rate of osteoporosis during OCP use, whereas a decreased HR was seen in women who were previous users, HR = 0.86 (0.83–0.89), compared to never users. In agreement with the largest fraction of osteoporosis diagnoses occurring in postmenopausal women (Fig. 1 C), we noted that only 3.7% (*n* = 659) of osteoporosis cases were diagnosed during OCP use.

**Table 3:** Effect of OCP during and after use on the risk of osteoporosis

Time-dependent associations	HR (95%) CI^a^	*N* ^b^	Person years	*p*-value
Never user	1 (Reference)	5 456	8 029 807	
During OCP use	1.00 (0.91–1.08)	659	2 554 587	0.84
Previous OCP use	0.86 (0.83–0.89)	11 658	6 243 868	7.0e-18

Hazard ratios (HR) from Cox regression analyses for the time-dependent association between OCP use and osteoporosis

*N* number of events, *HR* hazard ratio,* CI* confidence interval

^a^ Adjusted for year of birth, menopausal status, hysterectomy, bilateral oophorectomy, number of births, BMI and smoking status. Menopausal status, hysterectomy, bilateral oophorectomy and smoking were modelled as time-varying covariates and age was used as the primary timescale

^b^ Sample size after excluding individuals with missing data on the exposure or any of the covariates

## Discussion

Using a large population-based cohort, we show that OCP use is associated with higher BMD and reduced risk of osteoporosis later in life. The effect of OCP use on BMD is evident in postmenopausal women but not detected in premenopausal women. This might reflect a lack of power in a smaller dataset, but it is also possible that other mechanisms are of greater importance for BMD in premenopausal women or that the effect of OCP use differs with age and menopausal status.

The potential role of exogenous hormones administered during premenopausal years on skeletal health is intriguing but challenging to study. Few studies have a sufficiently long follow-up to assess effects that may appear decades after the exposure. The UKB cohort was collected in a cross-sectional manner, including women close to menopause, and BMD was recorded at recruitment [30]. Data regarding OCP use were collected retrospectively and register data on diagnosis of osteoporosis were collected both retro- and prospectively [24], making UKB a unique cohort for studying long-term effects of early/mid-life exposure on later life health effects. We analyze two phenotypes: BMD recorded by UKB and diagnosis of osteoporosis collected from multiple sources unrelated to the BMD data. The agreement between the two analyses, based on independently ascertained outcomes, strengthens our conclusion.

There are several limitations related to the information available in the UKB including that the type of OCP is unknown. However, given the birthyear distribution, we expect that the majority would have used first and second generation OCPs, which contain a combination of estrogen and progstin [31]. To account for cohort effects, which could influence the type of OCP used, we included year of birth as a covariate. Another limitation is that the duration of use was estimated based on reported first and last use without information on continuous use. It is probable that we have a bias towards null for the longer duration groups. Osteoporosis is often undiagnosed, and although we gathered data on osteoporosis diagnosis from multiple sources, the total number of women diagnosed with osteoporosis in the study underestimates the true prevalence. The net result of the aforementioned limitations would be a diluted signal and reduced statistical power, suggesting that our results underestimate the true effects. Since women were not randomized to receive treatment, there is a risk of confounding by indication. However, OCP use has not been considered a risk factor for osteoporosis, and no clinical recommendations suggest that high-risk individuals should avoid OCP use. Therefore, we judge that the risk of such bias inflating our results to be low.

Our analysis does not include other medications that could influence bone strength such as bisphosphonates, steroids, selective estrogen receptor modulators and HRT. The available data regarding such medications in this cohort is limited. To introduce such variables without careful consideration could cause collider bias [32], since, for example, HRT might be more often prescribed to women with a history of osteoporosis. Sample selection bias exists, as UKB participants are a healthier population compared to the UK general population [33], restricting generalizability. Nevertheless, in comparison with representative cohort studies, close agreement of risk factor associations has been shown [34]. Dual-energy X-ray absorptiometry (DXA) is considered the gold standard for evaluating BMD. In the current study, data from QUS were used. QUS does not involve radiation and provides a feasible alternative for screening large cohorts. Although it has been suggested that information obtained by QUS is comparable to that obtained by DXA [35, 36], our findings warrant confirmation in future studies with DXA data.

Osteoporosis is a silent disorder, often undiagnosed until a fragility fracture occurs. It is estimated that osteoporosis results in 1.5 million fractures annually in the United States [37] and 3.5 million in the European Union [38]. Osteoporotic fractures are associated with individual suffering and increased mortality as well as substantial costs for healthcare and society. Most fragility fractures occur in postmenopausal women, a fact usually attributed to reduced levels of female sex hormones [12]. Many women use exogenous hormones as contraceptives, and this could influence BMD and consequently later risk of osteoporosis. The current results are in line with previous studies suggesting a beneficial effect of OCP use on BMD in perimenopausal women [19, 20], but the beneficial effect of OCP use during the premenopausal years on skeletal health after menopause has not been shown before.

It has been discussed whether adolescent use of OCPs decreases long-term skeletal health and increases the risk of fragility fractures [39]. The rate of bone build-up peaks during puberty, around age 12 in girls of European ancestry [40]. Four years after the peak in bone accretion, 95% of adult bone mass is present [41]. Peak bone mass occurs by the end of the second or the beginning of the third decade of life, depending on the skeletal site. In our data, where most women were postmenopausal, the increase in BMD associated with OCP use was present even in women who started using OCP as adolescents, and our results did not confirm that adolescent OCP use increases the risk of osteoporosis later in life.

The increased BMD, as well as the reduced risk of osteoporosis demonstrated in postmenopausal women who used OCPs in their fertile years, suggests that the beneficial effects of OCP on skeletal health are detectable after use and are long-lasting. It has been estimated that around 80% of women of fertile age in North America and Europe will have used OCPs at some point [14], which corresponds well with the UKB cohort, where 82% of women reported ever having used OCP. Our results are therefore of significant importance to a large portion of the global population. However, in recent years, novel types of progestin-only contraceptives have become more common. As such, it is crucial to follow up on our findings as soon as sufficient follow-up time is available for these cohorts. This will allow us to assess whether progestin-only contraceptives potentially have fewer beneficial effects on bone health, which could lead to a significant increase in fractures among older women in the future.

## Electronic supplementary material

Below is the link to the electronic supplementary material.

Supplementary Material 1

## Data Availability

The data in the current study is available from the UKB Resource and can be accessed by application.
