# Supplementary material for: Oral contraceptive use increases bone density and reduces the risk of osteoporosis
Source: Eur J Epidemiol. 2025 Jul 9;40(9):1123–31. doi: 10.1007/s10654-025-01273-2 (PMC12537589; doi:10.1007/s10654-025-01273-2)
Supplement: Supplementary file 1 — Supplementary Material 1 [file 10654_2025_1273_MOESM1_ESM.docx]

**Table S1**: Characteristics of study population at recruitment to UK Biobank, the timepoint when BMD measurements were assessed and covariates collected.

| Supplementary Table 1 |  |  |
| --- | --- | --- |
|  | **Full dataset** | **Has BMD measurement** |
| **Total n** | 257 185 | 146 012 |
|  |  |  |
| **Age at recruitment** |  |  |
| *range* | 39-71 | 39-71 |
| *median* | 58 | 58.00 |
| *mean* | 56.6 | 56.46 |
|  |  |  |
| **Year of birth** |  |  |
| *range* | 1936-1970 | 1937-1968 |
| *median* | 1950 | 1950 |
| *mean* | 1951 | 1951 |
|  |  |  |
| **Menopausal status n(%)** |  |  |
| *pre menopause* | 58 455 (22.7%) | 33 645 (23.0%) |
| *post menopause* | 157 722 (61.3%) | 88 803 (60.8%) |
| *uncertain* | 40 708 (15.8%) | 23 401 (16.0%) |
| *missing data* | 300 (0.12%) | 163 (0.1%) |
| *Hysterectomy* | 48 536 (18.9%) | 27 823 (19.1%) |
| *Bilateral oophorectomy* | 20 752 (8.1%) | 11 440 (7.8%) |
|  |  |  |
| **Number of life births** |  |  |
| *range* | 0-22 | 0-22 |
| *median* | 2.00 | 2.00 |
| *mean* | 1.81 | 1.83 |
| *missing data* | 201 | 105 |
|  |  |  |
| **BMI (body mass index)** |  |  |
| *range* | 12.1-74.7 | 12.1-74.7 |
| *median* | 26 | 26 |
| *mean* | 27.0 | 27.0 |
| *missing data* | 1 041 | 195 |
|  |  |  |
| **Current smoking n(%)** | 23 086 (9.0%) | 12 925 (8.9%) |
| *missing data* | 143 | 78 |
|  |  |  |
| **Osteoporosis diagnosis (until end of follow up: 2020)** | 19 485 (7.6%) | 10 960 (7.5%) |
|  |  |  |
| **Oral contraceptives** |  |  |
| *never user* | 46 215 (18.0%) | 26 263 (82.0%) |
| *ever user* | 210 437 (82.0%) | 119 464 (18.0%) |
| *missing data* | 533 | 285 |
